
JOURNAL INFORMATION
==============================
NLM Title Abbreviation: ScientificWorldJournal
Journal ID: ScientificWorldJournal
NLM Journal ID: 101131163
Journal ID: TSWJ

The Scientific World Journal

ISSN: 2356-6140
EISSN: 1537-744X
Publisher: Wiley

ARTICLE INFORMATION
==============================
PMCID: PMC6083984
PMID: 12806062
DOI: 10.1100/tsw.2001.79
Article ID: 219295
Article version: 1
Subjects: Front Matter

Proceedings of the International Symposium on Passive Sampling of Gaseous Air Pollutants in Ecological Effects Research: Preface

Bytnerowicz Andrzej
Krupa Sagar
Cox Roger
USDA Forest Service, Pacific Southwest Research Station, Riverside, CA, USA
Publication date: 2001
Electronic publication date: 2001 Sep 18
Volume: 1
First page: 461
Last page: 462
Copyright: Copyright © 2001 Andrzej Bytnerowicz et al.
Copyright year: 2001
License: This is an open access article distributed under the Creative Commons Attribution License, which permits unrestricted use, distribution, and reproduction in any medium, provided the original work is properly cited.
License URL: https://creativecommons.org/licenses/by/3.0/

==============================
Preface
Proceedings of the International Symposium on Passive Sampling of Gaseous Air Pollutants in Ecological Effects Research
*TheScientificWorld* (2001) 1, 461–462
ISSN 1532-2246; DOI 10.1100/tsw.2001.79

### Proceedings of the International Symposium on Passive Sampling of Gaseous Air Pollutants in Ecological Effects Research: Preface

***Mission Inn, Riverside, CA***
***April 9, 2001***

**_Editors_**
Andrzej Bytnerowicz[1], Sagar Krupa[2], and Roger Cox[3]

[1]*USDA Forest Service, Pacific Southwest Research Station, Riverside, CA, U.S.*
[2]*Department of Plant Pathology, University of Minnesota, St. Paul, MN, U.S.*
[3]*Natural Resources Canada - Canadian Forest Service, Atlantic Forestry Centre, Fredericton, NB, Canada*

Some 150 years ago passive sampling was commenced at more than 300 stations to determine the relative concentrations of atmospheric ozone.* Since then, passive samplers such as sulfation candles and badge-type absorbents have been used to monitor point source emissions and pollutant concentrations in the work environment of industrial settings. Currently passive samplers are used in assessing human health–related exposures to air pollutants, compliance with regulatory requirements, industrial source performance such as leaks, and the efficacy of emission reduction strategies employed.

During the last decade there has been a rapid increase in the use of passive samplers for quantifying the ambient concentrations of gaseous air pollutants in ecological effects research. Since such samplers are inexpensive, easy to use, and do not require electrical power to operate, they have attracted wide interest in regional scale assessment of air quality. In the U.S., passive samplers have been used in a number of National Parks and in pristine wilderness areas. Their use is growing rapidly in developing countries as well.

In addition to use in U.S. National Parks, application of networks of passive samplers in vegetation effects studies includes the Frazer Valley, British Columbia and the Province of New Brunswick (Canada), Krakow region, the Sumava and the Carpathian Mountains (Europe), and the Sierra Nevada and the Cascade Mountains (U.S.). Both the Commission of the European Communities (CEC) and the North American Forestry Commission (NAFC) already have or have under consideration the deployment of passive sampling networks in assessing forest health.

The science of passive sampling of gaseous pollutants and relating the information to plant responses requires interdisciplinary collaboration among atmospheric scientists, meteorologists,

* Houzeau, A. (1858) Preuve de la présence dans l'atmosphère d'un nouveau principe gazeux, l'oxygène naissant. *C.R. Acad. Sci. Paris* **46**, 89–91.

©2001 with author.

plant scientists, and statisticians and/or modelers. Therefore, the international symposium held in Riverside, California provided an opportunity for some 100 participants with diverse backgrounds to meet and exchange information on the subject matter. The topics included: (a) development of passive samplers, (b) considerations for using passive samplers in field research, (c) case studies, and (d) development of risk assessment models based on landscape level pollution monitoring. Clearly much progress is being made in advancing the passive sampler technology, characterizing the regional scale air quality, and improving the utility of such data in relating to stochastic plant response relationships. Thus, we hope that the papers included in these conference proceedings prove to be of value in furthering our knowledge of the subject matter.

**Andrzej Bytnerowicz**
**Sagar Krupa**
**Roger Cox**
*September 18, 2001*

**This article should be referenced as follows:**

Bytnerowicz, A., Krupa, S.V., and Cox, R. (2001) Proceedings of the international symposium on passive sampling of gaseous air pollutants in ecological effects research: preface. *TheScientificWorld* 1, 461–462.
